# 325 An Ethnographic Exploration of Healthcare Professionals’ Interactions with Sinks and Water During Patient Care in Intensive Care Units

**DOI:** 10.1017/ash.2026.10671

**Published:** 2026-06-23

**Authors:** Allison Reeme

**Affiliations:** 1 Acacia Safety Consulting

## Abstract

**Background:** The rapid clinical translation of human gene therapy products, particularly those utilizing replication-deficient viral vectors such as adenovirus and adeno-associated virus, has introduced novel biosafety considerations regarding "shedding"—the dissemination of the vector through patient urine, saliva or stool. While therapeutic protocols focus on patient outcomes, unintentional exposure of healthcare workers, caregivers, or other close contacts to these viral vectors represents a critical, yet under-analyzed, epidemiological event. Objective: To critically evaluate the hazards posed by accidental exposure to recombinant viral vectors from an epidemiological perspective, focusing on risks of horizontal transmission and immunological interference. Discussion: Although modern viral vectors are engineered for safety, unintentional exposure via mucosal contact or accidental inoculation poses multiple risks that warrant critical evaluation. First, immunological priming or seroconversion in an exposed individual can induce development of neutralizing antibodies against the specific vector capsid. Such "silent" immunization may preclude an individual from receiving future gene therapies, creating a long-term public health barrier. Second, while rare, the risk of in vivo recombination with wild-type viruses (e.g., a natural adenovirus infection) could theoretically result in the rescue of replication-competent viral variants. Third, the biodistribution and persistence of a transgene in non-target populations remains poorly characterized. Identifying and tracking these events is essential for refining risk to public health, the environment and development of adequate containment guidelines for human gene therapy treatments utilizing viral vectors. **Conclusion:** Unintentional exposure to recombinant viral vectors represents a significant, under-recognized epidemiological hazard. To ensure the safe expansion of genetic medicine, it is imperative to move beyond patient-centric monitoring and treat unintentional exposures as epidemiological data points. Systematic surveillance of these events is vital for establishing a real-world safety profile of genetic medicines, refining biosafety protocols and safeguarding public health.

